# Network pharmacology, experimental validation and pharmacokinetics integrated strategy to reveal pharmacological mechanism of goutengsan on methamphetamine dependence

**DOI:** 10.3389/fphar.2024.1480562

**Published:** 2024-11-28

**Authors:** Han-Cheng Li, Jie-Yu Li, Xing-Chen Wang, Ming Zeng, Yang-Kai Wu, Yi-Ling Chen, Cai-Hua Kong, Ke-Lin Chen, Jie-Ru Wu, Zhi-Xian Mo, Jia-Xuan Zhang, Chang-Shun Liu

**Affiliations:** ^1^ Department of Pharmaceutical Engineering, School of Food and Pharmaceutical Engineering, Zhaoqing University, Zhaoqing, China; ^2^ Guangdong Provincial Key Laboratory of Chinese Medicine Pharmaceutics, School of Traditional Chinese Medicine, Southern Medical University, Guangzhou, China; ^3^ Risk Assessment Laboratory for Agricultural Product Quality and Safety, Ministry of Agriculture and Rural Development, Zhaoqing University, Zhaoqing, China; ^4^ Guangdong Basic Research Center of Excellence for Integrated Traditional and Western Medicine for Qingzhi Diseases, Guangzhou, China

**Keywords:** goutengsan, methamphetamine dependence, MAPK pathway, network pharmacology, pharmacokinetics

## Abstract

**Background:**

Goutengsan (GTS) is a traditional Chinese medicine formula that can improve multiple nervous system diseases, such as methamphetamine (MA) dependence. However, the mechanism how GTS treats MA dependence remains unclear. This study was aimed to investigate the action mechanism of GTS on MA dependence using network pharmacology, *in vivo*/*in vitro* experimental validation, pharmacokinetics, and tissue distribution in the brain.

**Materials and Methods:**

The bioactive ingredients from GTS and possible targeted genes for treating MA dependence were predicted using network pharmacology. The binding of key components of GTS to the predicted proteins was studied using molecular docking, and the key components were verified by HPLC. The effects of GTS on an MA-induced model in rats and SH-SY5Y cells were studied. The regulatory effects of GTS on the expressions of predicted MAPK pathway-related proteins in rat brain tissues and SH-SY5Y cells were validated. Furthermore, the plasma exposure and brain tissue distribution of GTS ingredients for MA dependence treatment and MAPK pathway regulation were studied in mice.

**Results:**

Network pharmacology screened 53 active ingredients, and 287 potential targets of GTS, and showed the MAPK pathway was among the most relevant pathways. Molecular docking showed that key active ingredients (e.g., 6-gingerol, liquiritin and rhynchophylline) bound strongly with MAPK core targets, such as MAPK3, and MAPK8. Five compounds of GTS were detected by HPLC, including 6-gingerol, chlorogenic acid, liquiritin, 5-o-methylviscumaboloside and hesperidin. GTS had a therapeutic effect on MA-dependent rats, and reduced hippocampal CA1 damage and relative expressions of p-MAPK3/MAPK3, p-MAPK8/MAPK8 in brain tissues induced by MA. GTS counteracted aberrant alterations in cAMP, 5-TH and cellular morphology induced by MA induction and exerts therapeutic effects on MA-induced SH-SY5Y cell models. GTS also can antagonize the high expressions of MAPK-related proteins in MA-induced SH-SY5Y cells. Pharmacokinetic experiment revealed the four ingredients of GTS (e.g., chlorogenic acid, 5-o-methylviscumaboloside, hesperidin and rhynchophylline) were exposed in the plasma and brain, which demonstrates its pharmacological effect on MA dependence.

**Conclusion:**

GTS treats MA dependence by regulating the MAPK pathway via multiple bioactive ingredients. The network pharmacology, experimental validation and pharmacokinetics integrated strategy is efficient in discovering the key pharmacological mechanism of herbal formulae.

## 1 Introduction

Drug dependence (DDp), as a chronic relapsing brain disease, is a hot and complex topic of medical research worldwide. Methamphetamine (MA) is among the most common new drugs in the world (Pastuzyn et al., 2012). The mainstream view on the mechanism of MA dependence is the dopamine theory, and the existing research on its mechanism is focused on the central nervous system (CNS) (Namyen et al., 2020). In addition to producing substantial CNS toxicity, drugs also have a severe damaging effect on the brain periphery. The liver, as the largest digestive gland in the body, is the center of the enrichment, transformation and metabolism of foreign chemicals including MA. MA enters the body and targets at hepatocytes, inducing liver damages (Golchoobian et al., 2017; Wang et al., 2017). Since there is no effective drug or method for MA dependence, it is urgent to find and explore effective therapeutic medicine or techniques to treat MA dependence.

Traditional Chinese medicine (TCM) for drug detoxification was already used at the end of the 19th century in China, and the most widely-used ones at that time were smoking cessation pills and tonic pills. To date, various TCM drug compound preparations have been approved for production and use in clinical treatment in China, such as Fukang tablets and Drug detoxification capsules. TCM for drug treatment is effective in treating delayed symptoms and preventing relapse, and most of the treatments are mild, less toxic, safe and non-addictive. Goutengsan (GTS) is a classic formula recorded in *Pu Ji Materia Medica* in 1132 A. D (Song et al., 2018). GTS contains 11 herbs (Table 1). Specifically, Uncariarhynchophylla, Dendranthema morifolium, and divaricate Saposhnikovia roots clear heat, calm the liver, quench wind, and stop spasms. Pinellia Ternata, Wolfiporia cocos, Citri Reticulatae Pericarpium, Glycyrrhiza uralensis Fisch, and Zingiber officinale Roscoe resolve phlegm, calm the heart, subdue rebellion, and stop vomiting. Moreover, raw gypsum, ginseng, and maitong clear heat, nourish yin, benefit qi, and produce fluids. Collectively, GTS can treat liver syncope and dizziness, and thus helps treat MA dependence. GTS can improve central nervous function, resisting oxidants and regulating blood pressure, and can be used clinically to treat Alzheimer’s disease (AD), memory disorders, cerebral infarction, and hypertension (Sasaki-Hamada et al., 2017; Zhao et al., 2005). Importantly, our previous studies found that Gou Teng (GT) and its bioactive ingredient rhynchophylline had good potential application in treating new synthetic drug dependence by regulating neurotransmitter levels, glutamate receptors, and neuroprotection (Li et al., 2023; Li et al., 2018a; Zhou et al., 2020). The pharmacology and clinical application of GTS suggest it has potential therapeutic effects on DDp, but there is no report on the treatment of DDp with GTS.

**TABLE 1:** Details about the formula of GTS.

Herbal components	Traditional Chinese name	Scientific name	Family of the plant	Plant part used	Ratios in GTS (%)
Chrysanthemum	Ju Hua	Chrysanthemi Flos	Asteraceae	Capitulum	6.67
Liquorice Root	Gan Cao	Glycyrrhizae Radix et Rhizoma	Leguminosae	Roots and rhizome	3.33
Divaricate Saposhniovia Root	Fang Feng	Saposhnikoviae Radix	Umbelliferae	Roots	6.67
Tangerine Peel	Chen Pi	Citri Reticulatae Pericarpium	Rutaceae	Pericarp	10.00
Ginger	Sheng Jiang	Zingiberis Rhizoma	Zingiberaceae	Rhizome	3.33
Gambir Plant	Gou Teng	Uncariae Ramulus cum Uncis	Rubiaceae	Stems and branches	10.00
Pinellia Ternata	Ban Xia	Pinelliae Rhizoma	Araceae	Tubers	10.00
Dwarf Lilyturf Tuber	Mai Dong	Ophiopogonis Radix	Liliaceae	Tuberous roots	10.00
Indian Buead	Fu Ling	Poria	Polyporaceae	Mycorrhizae	10.00
Ginseng	Ren Shen	Ginseng Radix et Rhizoma	Araliaceae	Roots and rhizomes	23.33
Gypsum	Shi Gao	Gypsum Fibrosum	Sulfates	Minerals	6.67

Network pharmacology is a novel technological term integrating pharmacological data, omics, and systems biology. According to this concept, drugs target multiple nodes in interconnected systems (rather than individual nodes), which generate information for drug assessment (Boezio et al., 2017). The idea of network pharmacology is consistent with the action mechanism of TCM, which emphasizes synergistic effects and interrelation. TCM formulae are composed of several herbs and compounds exerting synergistic effects by affecting multiple genes, proteins, and pathways. Network pharmacology can explain the interaction between multiple compounds and disease targets, and allows to explore the actions of TCM (Li and Zhang, 2013). Network pharmacology of TCM is widely studied (Li et al., 2020; Liang et al., 2020). However, most studies only focus on the individual ingredients rather than the contents or *in vivo* pharmacokinetic characteristics of these ingredients. Pharmacokinetic study reveals the absorption efficiency, distribution tendency, and biological stability of a drug, and demonstrates its bioactivity on the disease. Sufficient drug concentration at the target site of the disease is a prerequisite for its pharmacological activity (Chen et al., 2019).

The integration of network pharmacology with pharmacokinetics offers a robust strategy to address the limitations of each individual approach, enabling a comprehensive and precise understanding of the pharmacological mechanisms in TCM formulations (Wu et al., 2022). For instance, pharmacokinetic data provide network pharmacology with more accurate quantitative information on the active ingredients, thus reducing the likelihood of overlooking low-abundance compounds with significant bioactivity (Li et al., 2021). Conversely, network pharmacology supplies pharmacokinetics with valuable insights into the target pathways of key constituents, enabling the study to focus on components most impactful to therapeutic efficacy, and minimizing redundant experimental designs (Tang et al., 2022). This synergy not only enhances research efficiency but also improves the accuracy and scientific rigor of the findings. Thus, the network pharmacology and pharmacokinetics integrated strategy is an effective method to reveal the pharmacological mechanism of TCM formulas.

**FIGURE 1:**
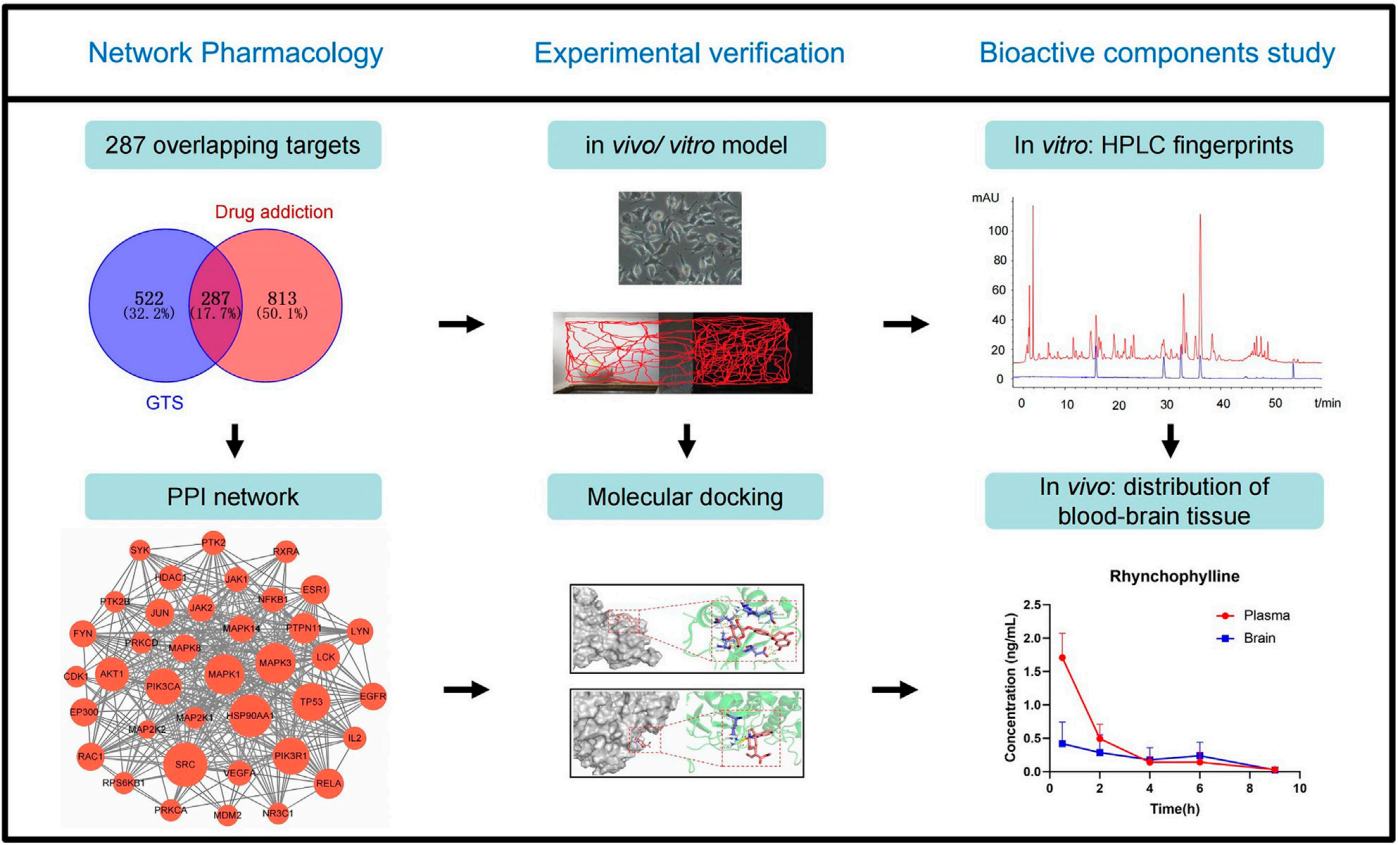
Workflow diagram of this study.

In this study, the bioactive ingredients from GTS and the possible targeted genes for treating MA dependence were predicted using network pharmacology and molecular docking. The effects of GTS on an MA-induced model in rats and SH-SY5Y cells were studied, and the predicted MAPK pathway was verified. Moreover, the plasma exposures and brain tissue distribution of the bioactive ingredients from GTS were studied in mice. This study will help further clarify the material basis and possible mechanism of action of GTS against MA (Figure 1).

## 2 Materials and Methods

### 2.1 Chemicals and biological materials

Methamphetamine hydrochloride (size: 20 mg per vial; purity ≥98%; lot number 1212-9802) was purchased from the National Narcotics Laboratory of China. MK-801 was bought from Sigma (St. Louis, MO, United States; lot number 022M4616V). Sterile physiological saline was offered from Guangdong Kellen Pharmaceutical Co. Ltd (Approval No., State Pharmacopoeia H20046295). Conditioned place preference (CPP) black and white box were obtained from Wuhan Yihong Technology Co., Ltd (China). A rat CPP behavioral analysis system was purchased from Noldus, Information Technology, Wageningen, Netherlands. SH-SY5Y cells were provided by the Cell Resource Center of Shanghai Institute of Life Sciences, Chinese Academy of Sciences. RPMI 1640 medium, fetal bovine serum, heat-inactivated horse serum, trypsin, penicillin and streptomycin were provided by Gibco (Grand Island, United States). SYBR^®^ PrimeScript miRNA RT-PCR Kit was purchased from TaKaRa (Dalian, China). MiRNeasy Mini Kit was purchased from QIAGEN (Hilden, Germany). Dual-luciferase assay kit was provided by Promega (Madison, United States).

### 2.2 Network pharmacological experiment

#### 2.2.1 Related targets of DDp and ingredients of GTS

Active ingredients of GTS were obtained from TCMSP (http://lsp.nwu.edu.cn/tcmsp.php) (Ru et al., 2014). Based on the characteristics of GTS tonics in oral form, the relevant parameters for screening were set as follows: OB% (oral bioavailability) ≥30, DL (drug-likeness) ≥0.18, BBB (blood–brain barrier) ≥0.3 (Zhang et al., 2019). The names of the resulting active ingredients in GTS were entered into Pubchem (https://pubchem.ncbi.nlm.nih.gov/), corresponding to the names and molecular mass numbers of the compounds obtained from the search. Then the names were inputted into the Swiss target prediction database (https://swisstargetprediction.ch/), and the corresponding targets for the compounds were exported. Search was conducted using keywords “Drug addiction” and “Drug dependence” on DisGeMET (https://www.disgenet.org/dbinfo) and GeneCards (https://www.genecards.org/) (Barshir et al., 2021). All the searched targets were integrated and de-weighted, and Relevance score ≥10 was set as a filter condition for DA targets. Venn diagrams of interactions between GTS-related targets and DDp targets were plotted using Venny 2.1 (https://bioinfogp.cnb.csic.es/tools/venny/).

#### 2.2.2 Construction of multiple network viewable

The active ingredients of GTS and their corresponding targets were organized into a network file, and the data in the network file were tagged with categories in the tape file. Herbs-compounds-targets visual network diagrams were constructed on CytoScape 3.9.1.

#### 2.2.3 GO and KEGG enrichment analysis

GO and KEGG enrichment analysis was performed with the common genes of GTS-DDp on DAVID (https://david.ncifcrf.gov/) (Sato et al., 2020). Three sections of the GO analysis were used to annotate the function of target proteins from the perspectives of biological process (BP), cellular component (CC), and molecular function (MF). GO and KEGG enrichment maps were plotted using a microbiology letter platform.

#### 2.2.4 Construction of protein-protein interaction (PPI) network and analysis of cluster module

The PPI network diagram of GTS-DA was obtained from TRING (https://string-db.org/), a searchable online database of known protein interactions (Szklarczyk et al., 2015). Cluster modules for network graphs were constructed using the plugin MCODE to more visually show the drug - disease association and to facilitate the next step of analysis.

### 2.3 Molecular docking

Protein structures of core targets as macromolecular rigid receptors were obtained through PDB (https://www.rcsb.org/), Uniprot (https://www.uniprot.org/), and TCMSP to obtain the structures of the core active ingredients of GTS as small molecules conformationally changeable flexible ligand (Berman et al., 2020). The file was formatted using OpenBabel and imported into AutoDock 4.2.6 for semi-flexible molecular docking of the receptor to the ligand (Mohamed A et al., 2019). The intermolecular free binding energy was used as a reference value to determine the superiority of the active ingredient binding to the target site. The free binding energy <0 proves the receptor and ligand are free to bind, and that ≤ −2 indicates the active ingredient is well-docked to the target site (Song et al., 2020; Clyne et al., 2020). Finally, the composition of the better paired component-target combinations was visualized on PyMOL (Delano, 2002; Jerine et al., 2021).

### 2.4 Preparation, fingerprint analysis and quantification ingredients of GTS

According to the composition of the prescription ratio of GTS (Table 1), the concentrated extract was obtained using a water decoction method. Specifically, Ginger, Divaricate Saposhniovia Root, Tangerine Peel and Chrysanthemum were separately extracted with volatile oil, and mixed with the above concentrated extract, forming an infusion of 2.75 g of crude drug/mL. The infusion of GTS was ground and weighed (1.0 mL), and added with 5 mL of acetonitrile-water (5:5, v/v) under stirring. Then the samples were placed in an ultrasound machine for 50 min of extraction, and centrifugated to obtain supernatants. After dilution (1.5 mg/g crude drug), a supernatant aliquot of 10 μL was analyzed by HPLC. The HPLC system consisted of an Agilent 1290 liquid chromatograph and a DAD ultraviolet detector (Agilent Technologies Inc., CA, United States). The analytes were eluted using an ACE Excel 3 C_18_ column (2.1 mm × 100 mm, 3 μm). The column and autosampler was set at 25°C. Samples were separated with a mobile phase consisting of A (acetonitrile) and B (50 mM KH_2_PO_4_). The solvent gradient was 0–12 min with 20% A; 12–20 min with 20%–30% A; 20-30 min with 70% A. The flow rate was 1.0 mL/min, and the detection wavelength was 225 nm. Five chemical constituents in the GTS were measured quantitatively using HPLC.

### 2.5 Experimental validation in rats with MA-dependent model

Male Sprague-Dawley (SD) rats weighing 180–220 g (2 months old) were provided by the Experimental Animal Center of Southern Medical University. The rats were maintained at room temperature (20 ± 2) °C, humidity (60 ± 5)%, 12 h light cycles, and provided an *ad libitum* diet. Before commencing the experiment, the rats were allowed to acclimatize for a minimum of 3 days. The Southern Medical University Experimental Animal Ethics Committee approved all animal experiments, which were conducted in accordance with the National Institutes of Health Guide for the Care and Use of Laboratory Animals (NIH publication #85–23, revised 1985). As a non-competitive antagonist of N-methyl-D-aspartic acid (NMDA) receptors, MK-801 (Dizocilpine) was utilised as a positive control drug because it was able to inhibit the CPP effect caused by MA in rats (Niikura et al., 2015), and MK-801 were used at 10 mg/kg by our preliminary test. An MA-dependent rat CPP model was established in our previous studies and MA was used at 2 mg/kg (Li et al., 2018b). Prepare solutions of 0.4 mg/mL MA, 2.0 mg/mL MK-801, and 2.2 g crude drug/mL GTS in physiological saline. Before administration, determine the dosing volume based on each rat’s body weight; for example, a 200 g rat would receive a 1 mL dose.

Forty qualified rats in the baseline test were randomly assigned to four groups: a control group, an MA model group (2 mg/kg), a GTS treatment group (11.00 g crude drug/kg), an MK-801 positive drug group (10 mg/kg). We investigated the effects of different doses of GTS and found that 11.00 g crude drug/kg (4x equivalent dose) GTS could significantly reverse MA-induced CPP effect in rats in our preliminary test. Given the scarcity and specificity of MA, we decided to use a 4x equivalent dose of GTS as the therapeutic dose. Thus, in the current study, only one treatment dose group of GTS was selected.

The CPP exam was made up of three phases over the course of 10 days (Figure 5A). During the habituation phase (days 1–3), each rat was allowed to move freely firom the black to white compartment for 30 min each day. On day 3, rats were videotaped throughout a 15-min trial and their movement trajectories, time spent and total movement distances were recorded. The door was closed to keep the two compartments apart during the conditioning period (on days 4–9). At 8:00 a.m. on days 4–9, rats received a subcutaneous injection (s.c.) of MA (2 mg/kg) once daily for 6 consecutive days in the MA model, GTS treatment and MK-801 positive drug groups or the same volume of physiological saline (s.c.) in the control group, after which they were immediately sequestered in the white compartment for 1 h. Rats were given the same amount of physiological saline (s.c.) in each group after an 8-h break, and they were then promptly segregated into the black compartment for 1 h. At 20:00 p.m. on days 5–9, the rats were intraperitoneally injection (i.p.) GTS (4x equivalent dose) and MK-801 (10 mg/kg) once daily for 5 consecutive days, respectively, in GTS treatment and MK-801 positive drug groups or the same volume of physiological saline (i.p.) in the control and MA model groups. At 0:00 a.m. on day 10 (testing phase), the formal CPP testing began, rats were videotaped throughout a 15-min trial using Noldus Ethovision XT 8.5 software, and their movement trajectories, time spent and total movement distances were recorded.

### 2.6 Histopathological observation

After the CPP test, a cervical dislocation method was used to euthanize the rats. The brain tissue of each rat was cut along the midline of the sagittal plane in the head. The paraffin coronal section of the rat brain showed hippocampus and dentate gyrus. The hippocampus consists of CA1, CA2, CA3, and CA4. Specifically, CA1 is mainly composed of small vertebral cells, and is more suitable for MA-dependent observation (Zhong et al., 2012). Therefore, the pyramidal cells in hippocampal CA1 were mainly observed after hematoxylin-eosin (HE) staining. The hippocampal CA1 after HE staining was observed under microscopy and then photographed.

### 2.7 SH-SY5Y cell experiment

#### 2.7.1 Preparation of GTS-containing serum

The mother serum liquor was set at a 16x equivalent dose (44.00 g of crude drug kg^−1^). Ten SD rats were selected and gavaged with 16x equivalent dose of the GTS extract twice a day for 3 consecutive days. Blood was collected. The serum was separated within 1 h of the last treatment and filtered through a 0.22 μm micropore membrane to obtain GTS-containing serum. Then the mother serum liquor was diluted sequentially to obtain 16x, 8x, 4x and 2x equivalent dose GTS-containing serums.

#### 2.7.2 MTT detection of cell viability

SH-SY5Y cells were inoculated into a 96-well plate. After the culture medium was aspirated, gradient diluted MA (0, 25, 50, 100, 200 μmol ·L^−1^) and GTS-containing serum (0, 2, 4, 8, 16x equivalent dose) were added at 100 μL each separately. After 48 h of incubation at 6 wells per concentration, MTT was added for 4 h. Then incubation was stopped, the liquid was discarded, and dimethyl sulfoxide was added. After horizontal shakening for 5 min, the absorbance at 490 nm was measured using an enzyme-linked immunosorbent assay (Elisa) reader (Yang et al., 2018).

#### 2.7.3 Cell experiments

SH-SY5Y cells were randomly divided into four groups: a control group, an MA model group (100 μmol ·L^−1^), a GTS treatment group (4x equivalent dose GTS-containing serum) and an MK-801 positive drug group (100 μmol ·L^−1^). An equal volume of the complete culture medium was added to the control group. The drug-containing culture medium (100 μmol ·L^−1^ MA) was added to MA model group. After incubation with a 4x equivalent dose of GTS-containing serum and a 100 μmol ·L^−1^ MK-801 for 15 min in GTS treatment and MK-801 positive drug groups, respectively, then 100 μmol ·L^−1^ MA drug-containing culture medium was added. After medication, all groups were continued for 48 h, photographed, and used for the next experiment.

#### 2.7.4 Elisa detection of cAMP and 5-HT

The above SH-SY5Y cells were inoculated onto a 6-well plate, and cultured for 24 h. Then the culture medium was aspirated. The cells were rinsed with phosphate buffered salt (PBS) once. After PBS was removed, a hydrochloric acid solution was added. The cells were scraped to form cell suspension. After centrifugation, the supernatant was extracted, and the cAMP and 5-HT levels of the cells were detected according to the instructions of an Elisa reagent kit (Dakewei Biotechnology Co., Ltd.) (Zhan et al., 2022).

### 2.8 Western blot

MAPK-related protein expressions in rat brain and SH-SY5Y cells were detected via Western blot (Harris, 2015; Tabei et al., 2023). An appropriate amount of brain tissue from each group was taken for homogenization, and added with 1 mL of a tissue lysis solution, protease inhibitor or phosphorylated protease inhibitor (1:100). Moreover, SH-SY5Y cells from each group were taken, cultured for 24 h, and added with cell lysate for sufficient reaction. Total proteins were extracted, and the protein concentration was measured in an ultra-micro spectrophotometer. The relative expressions of MAPK-related proteins (MAPK3, MAPK8, p-MAPK3, p-MAPK8) in rat brain tissues and SH-SY5Y cells were detected by Western blot. Enhanced chemiluminescence (ECL) was used for developing. Photos were taken using a ChemiDoc all-purpose gel imager, and gray-scale analysis was finished on ImageJ. Relative expression was computed as target protein grayscale value/GAPDH grayscale value.

### 2.9 Pharmacokinetics and tissue distribution analysis in mice

Animal experiments: Babl/c mice weighing 18–22 g (6–8 weeks old) with equal numbers of males and females, were provided by the Experimental Animal Center of Southern Medical University. The mice were maintained at room temperature (20 ± 2) °C, humidity (60 ± 5)%, 12 h light cycles, and provided an *ad libitum* diet. Thirty mice were randomly divided into 0.5, 2, 4, 6 and 9 h groups, with 6 mice each. An infusion at concentration of 2.75 g of GTS crude drug/mL was obtained, and the drug was administered by gavage at a dose of 2 mL GTS infusion drug/kg. After 0.5, 2, 4, 6 and 9 h of administration, the plasma and brain tissues in the 0.5, 2, 4, 6 and 9 h groups were collected. The plasma and brain tissue samples were processed in accordance with biological sample processing procedures.

Establishment of UPLC-MS method: An LCMS-8050 liquid chromatography mass spectrometer (Shimadzu, Japan) was operated with a horizon C_18_ column (100 × 2.1 mm, 3 µm). The mobile phase was 0.1% formic acid in water (A) - acetonitrile (B), and the gradient elution was 0–8 min, 10%–50% B; 8–9 min, 50%–10% B; 9–11 min, 10% B. The column temperature was 30°C, the injection volume was 5 μL, and flow rate was 0.4 mL min^−1^. The mass spectrometry conditions included HPLC-MS/MS in O_3_ full-scan and sub-ion scanning modes, an electrospray ionization source (ESI), positive and negative ion mode detection, and nebulizing gas of nitrogen (N_2_). The zeroed gas flow rate was 3 L min^−1^, desolventization temperature was 526°C, and DL temperature was 250°C. Then the parent ion, daughter ion, collision energy, and polarity of the above components were determined (Table 2).

**TABLE 2:** UPLC-MS conditions.

Chemical compound	Molecular mass	Parent ion (m/z)	Daughter ion (m/z)	Ce (eV)	Scanning mode
Chlorogenic acid	354.3	352.9	190.9	20.0	-
Liquiritin	418.4	416.9	254.9	21.0	-
5-O-Methylviscumaboloside	452.5	452.8	291.2	−25.0	+
Hesperidin	610.6	609.0	300.9	31.0	-
Rhynchophylline	384.5	384.9	160.2	−30.0	+
Glycyrrhizic acid	823.0	821.4	350.6	37.0	-

### 2.10 Statistical analysis

SPSS 22.0 (International Business Machines Corporation) and GraphPad Prism 8.0 (GraphPad Software Corporation) were used for statistical analysis. Data were expressed as mean ± SEM. The differences between groups were tested using one-way ANOVA analysis of variance. Testing for homogeneity of variance in inter-group comparisons was first carried out using Bonferroni’s test. If variances were unequal, the corrected F-test (Welch’s test) was used and Tamhane’s T2 test was used for multiple comparisons. A *p* < 0.05 indicated statistical significance.

## 3 Results

### 3.1 Prediction of possible targets and pathways of GTS in MA dependence treatment

**FIGURE 2:**
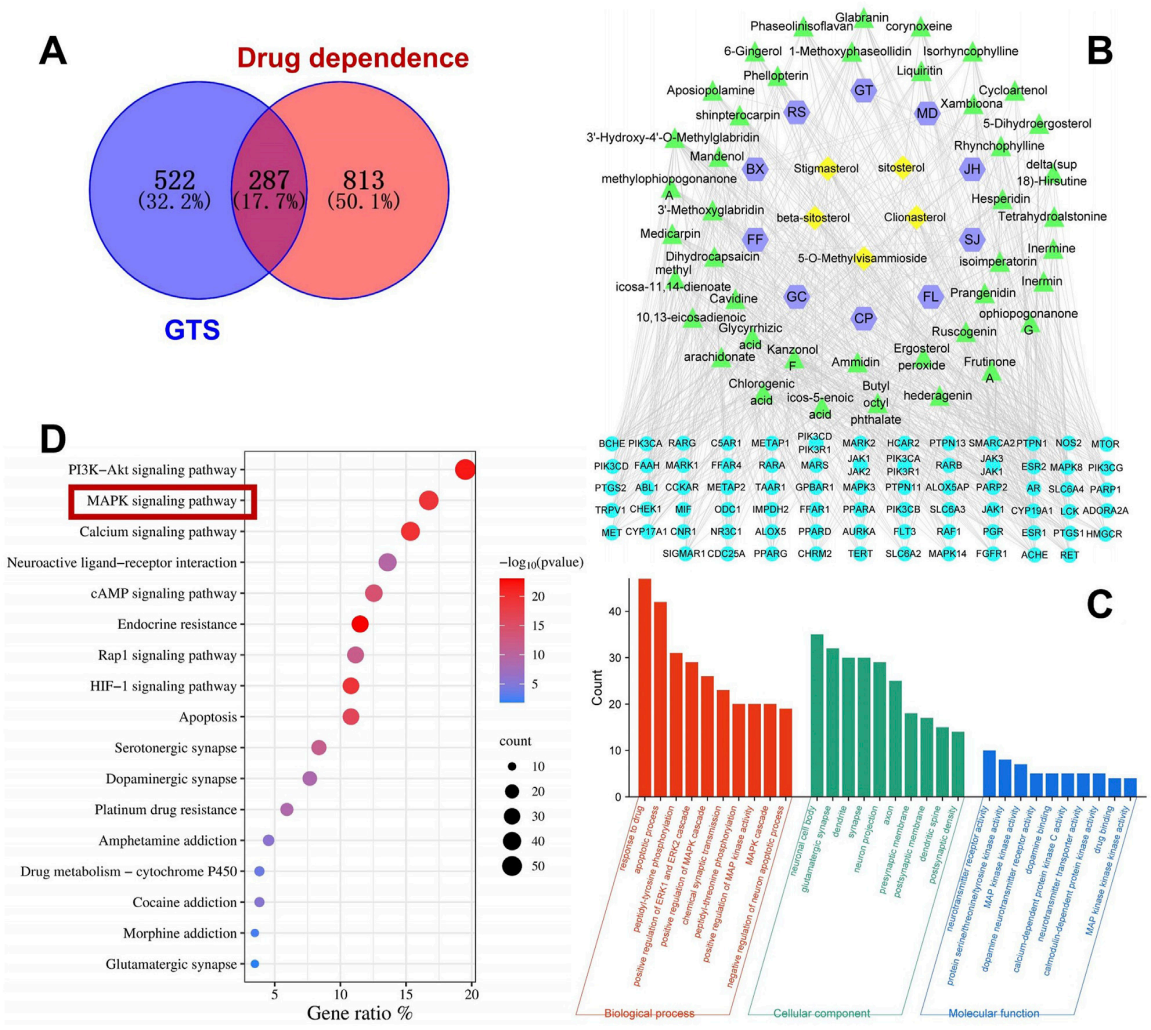
Network pharmacology analysis of GTS intervention in DDp. **(A)** Venn diagram of overlapping targets between drug-related targets of GTS and the targets associated with DDp. **(B)** Herbs-component-disease target network, JH, Ju Hua (Chrysanthemi Flos); GC, Gan Cao (Glycyrrhizae Radix et Rhizoma); FL, Fu Ling (Poria); FF, Fang Feng (Saposhnikoviae Radix); CP, Chen Pi (Citri Reticulatae Pericarpium); BX, Ban Xia (Pinelliae Rhizoma); SJ, Sheng Jiang (Zingiberis Rhizoma); RS, Ren Shen (Ginseng Radix et Rhizoma); MD, Mai Dong (Ophiopogonis Radix); GT, Gou Teng (Uncariae Ramulus cum Uncis); Shi Gao (Gypsum Fibrosum) is only predicted one major component, CaSO_4_, so it fails to be shown in the network. **(C)** GO enrichment analysis. **(D)** KEGG enrichment analysis.

Totally 53 active ingredients of GTS were obtained. Specific information can be found in Table 3. Analysis revealed 287 shared targets between the DDp targets and the active compounds of GTS (Figure 2A). These shared targets represent the key points of action for the active compounds of GTS in treating DDp. Then 53 active compounds for GTS treatment of DDp were paired with 287 intersecting targets, generating network and tape files, which were imported into Cytoscape. The correlations among herbs-ingredient-target were further improved, and a network with 173 nodes and 764 edges was constructed (Figure 2B). The targets with higher degrees include MAPK3, MAPK8, and MAPK14.

**TABLE 3:** Data of 53 active ingredients in Goutengsan.

Number	Mol Id	Main active ingredient(s)	OB%	BBB	DL
1	MOL008487	Hirsutine	34.44	0.78	0.43
2	MOL008457	Tetrahydroalstonine	32.42	0.33	0.81
3	MOL008489	delta (sup 18)-Hirsutine	41.64	0.76	0.64
4	MOL008465	Hesperidin	32.75	0.82	0.64
5	MOL008477	Corynoxeine	57.13	0.50	0.57
6	MOL008469	Rhynchophylline	41.82	0.38	0.57
7	MOL008471	Isorhyncophylline	47.31	0.33	0.57
8	MOL000358	Beta-sitosterol	36.91	0.99	0.75
9	MOL000359	Sitosterol	36.91	0.87	0.75
10	MOL002670	Cavidine	35.64	0.63	0.81
11	MOL006936	10,13-eicosadienoic acid	39.99	0.82	0.20
12	MOL003578	Cycloartenol	38.69	1.33	0.78
13	MOL000449	Stigmasterol	43.83	1.00	0.76
14	MOL005030	Glycyrrhizic acid	30.70	0.80	0.20
15	MOL001755	24-ethylcholest-4-en-3-one	36.08	1.22	0.76
16	MOL000282	5-dihydroergosterol	43.51	0.91	0.72
17	MOL000283	Ergosterol peroxide	40.36	0.34	0.81
18	MOL000296	Hederagenin	36.91	0.96	0.75
19	MOL005308	Aponorhyoscine	66.65	0.40	0.22
20	MOL003648	Inermin	65.83	0.36	0.54
21	MOL005321	Frutinone A	65.90	0.46	0.34
22	MOL005356	6-Gingerol	61.22	1.22	0.31
23	MOL005320	Arachidonate	45.57	0.58	0.20
24	MOL005399	Alexandrine-qt	36.91	0.88	0.75
25	MOL001771	Clionasterol	36.91	1.14	0.75
26	MOL011319	Butyl octyl phthalate	43.74	0.60	0.24
27	MOL011749	Phelloptorin	43.39	0.43	0.28
28	MOL002644	Phellopterin	40.19	0.48	0.28
29	MOL001941	Ammidin	34.55	0.92	0.22
30	MOL001942	Isoimperatorin	45.46	0.66	0.23
31	MOL003588	Prangenidin	36.31	0.50	0.22
32	MOL001494	Mandenol	42.00	1.14	0.19
33	MOL007514	Methyl icosa-11,14-dienoate	39.67	1.10	0.23
34	MOL001484	Inermine	75.18	0.40	0.54
35	MOL002565	Medicarpin	49.22	0.53	0.34
36	MOL003896	7-methoxy-2-methyl isoflavone	42.56	0.56	0.20
37	MOL004891	Shinpterocarpin	80.30	0.68	0.73
38	MOL004966	3′-hydroxy-4′-O-methylglabridin	43.71	0.73	0.57
39	MOL004974	3′-methoxyglabridin	46.16	0.47	0.57
40	MOL004833	Phaseolinisoflavan	32.01	0.46	0.45
41	MOL004908	Glabridin	53.25	0.36	0.47
42	MOL004959	1-methoxyphaseollidin	69.98	0.48	0.64
43	MOL004910	Glabranin	52.90	0.31	0.31
44	MOL005003	Liquiritin	58.81	0.61	0.58
45	MOL005018	Xambioona	54.85	0.52	0.87
46	MOL004988	5-O-Methylviscumaboloside	32.47	0.56	0.89
47	MOL004806	Euchrenone	30.29	0.39	0.57
48	MOL004985	Icos-5-enoic acid	30.70	1.09	0.20
49	MOL004996	Chlorogenic acid	30.70	0.94	0.20
50	MOL008698	Dihydrocapsaicin	47.07	0.47	0.19
51	–	Methylophiopogonanone A	–	–	–
52	–	Ophiopogonanone G	–	–	–
53	–	CaSO_4_	–	–	–

OB, oral bioavailability; BBB, blood–brain–barrier; DL, drug-likeness.

As shown in Figure 2C, in the BP analysis, the target genes were largely enriched in apoptotic process, positive regulation of MAPK cascade and chemical synaptic transmission; in the CC analysis, neuronal cell body, glutamatergic synapse, dendrite and synapse were shown to be involved, while in the MF analysis, neurotransmitter receptor activity, MAPK activity and dopamine neurotransmitter receptor activity were discovered to be involved. KEGG pathway enrichment analysis was performed on the 287 intersecting targets, and yielded totally 180 related pathways, mainly including PI3K-Akt, MAPK and cAMP signaling pathways (Figure 2D).

### 3.2 Screening for core targets of GTS intervention in DDp

**FIGURE 3:**
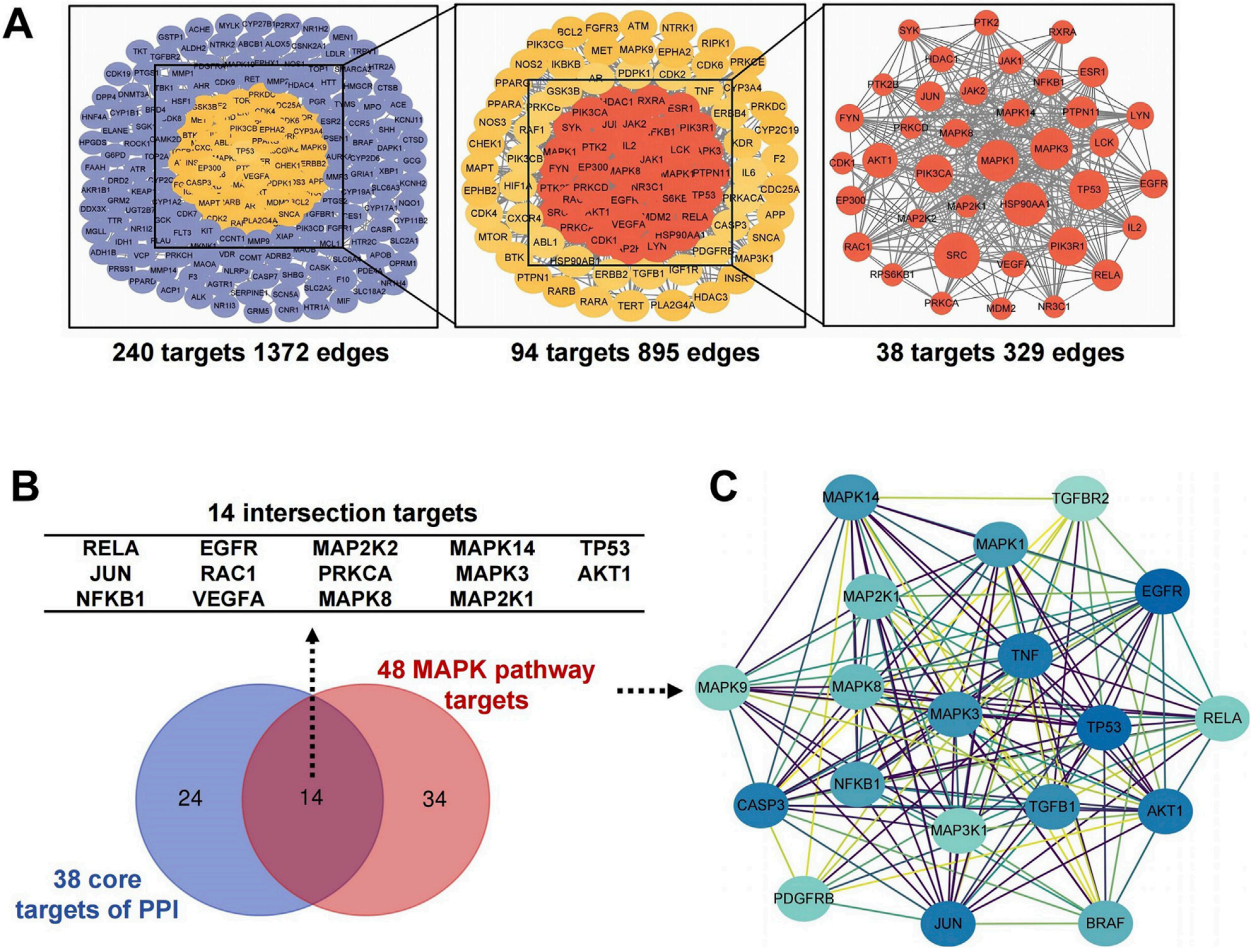
Screening for core targets of GTS intervention in DDp. **(A)** GTS key target PPI network. **(B)** Venny diagram of 14 intersection targets between 38 core targets and 48 MAPK pathway targets. **(C)** Cluster module of 48 MAPK pathway targets.

The targets were screened with a medium confidence of 0.9, which resulted in 240 targets and 1372 edges. The first screening with thresholds of Degree > 10.00, Between > 34.45, and Closeness>0.28 yielded 94 targets and 895 edges. The second screening with thresholds of Degree > 21.00, Between > 111.62, and Closeness > 0.38 identified 38 targets and 329 edges for screening analysis (Figure 3A). Among them, 14 targets with high degree, including MAPK14, MAPK8, and MAPK3, were all enriched in the MAPK pathway (Figure 3B). Hence, the MAPK pathway plays a crucial role in treating DDp with GTS.

Further screening and analysis were conducted on 583 intersecting targets, and 48 targets were found in the MAPK pathway. PPI network cluster module analysis with the 48 MAPK pathway targets was performed using the MCODE plugin in Cytoscape, resulting in a total of cluster modules (Figure 3C). Software-based analysis of data showed a protein network cluster was constructed with MAPK3 as the core target, and the core positions of the network were processed with MAPK1, MAPK8, MAPK14, MAP2K1 and other targets. Results suggest GTS may exert a therapeutic effect on DDp by regulating the above core targets in the MAPK pathway.

### 3.3 Validation of predicted targets and bioactive ingredients of GTS

**FIGURE 4:**
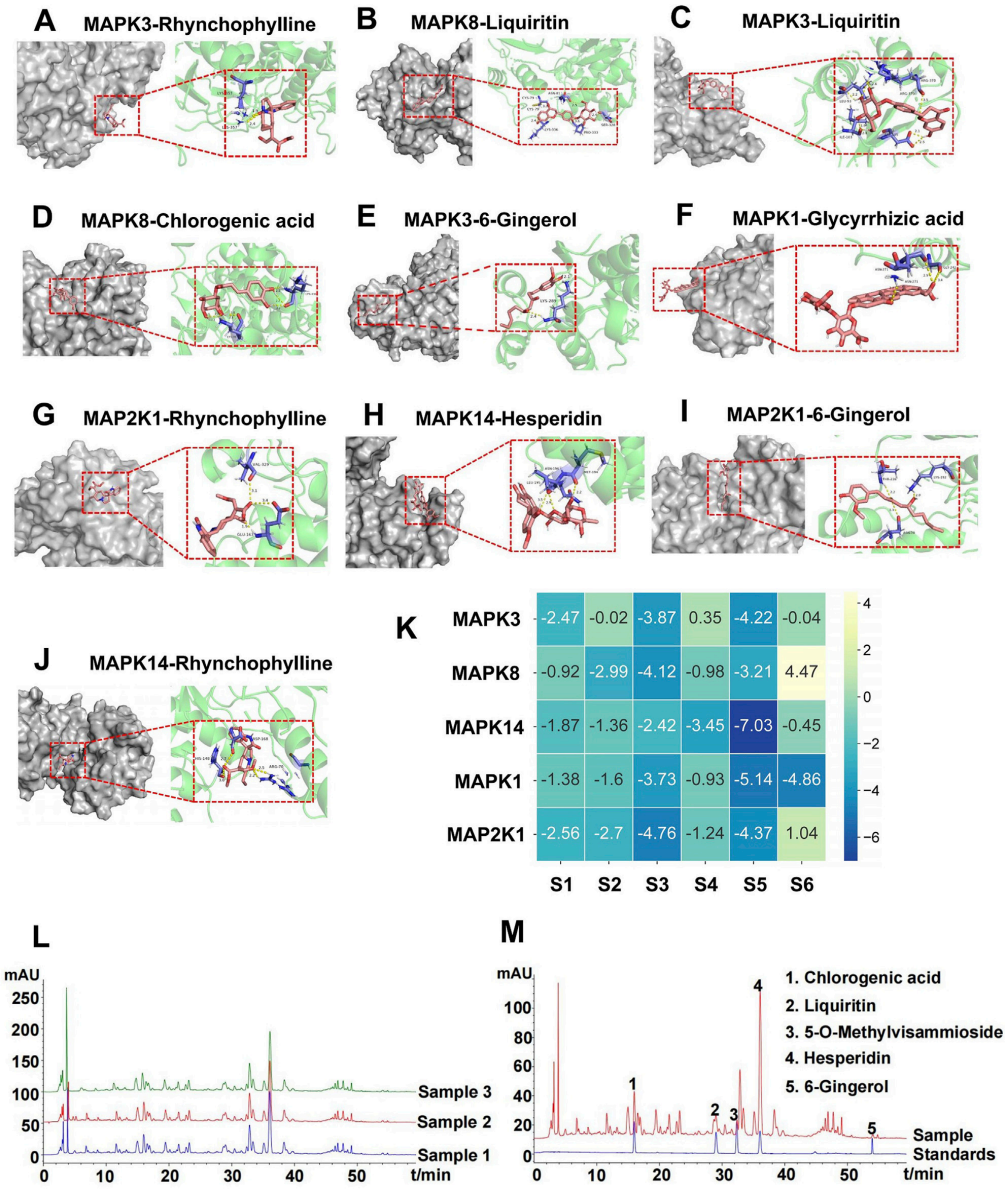
Molecular docking and representative HPLC-based chemoprofiles of GTS samples. Molecular docking between six active ingredients of GTS and five core targets in MAPK pathway **(A–J)**. **(K)** Combined energy of molecular docking between six active ingredients of GTS and five core targets in MAPK pathway, S1: 6-Gingerol, S2: Chlorogenic acid, S3: Liquiritin, S4: Hesperidin, S5: Rhynchophylline, S6: Glycyrrhizic acid. **(L)** Repeatability analysis of three GTS samples with HPLC fingerprints. **(M)** Five ingredients of GTS samples identified with HPLC fingerprints.

The key compounds of GTS for treating DDp were selected. Rhynchophylline, liquiritin, chlorogenic acid, glycyrrhizic acid, 6-gingerol, and hesperidin were chosen as ligand molecules, and the ligand molecule mol2 file was obtained through the TCMSP database. The corresponding molecular rigid receptors for the core targets MAPK3, MAPK8, MAPK1, MAP2K1 and MAPK14 of the MPAK pathway were retrieved through Uniprot, and the large molecular rigid receptor pdbqt file was downloaded. The obtained macromolecular rigid receptor files were preprocessed on PyMOL for molecular docking, and receptor ligand docking was finished on Auto Duck 4.2.6. The molecular docking images were presented using PyMOL (Figures 4A–J), indicating a stable interaction between the above active ingredients and the core target of the MAPK pathway. Combined energy of molecular docking between six active ingredients of GTS and five core targets in MAPK pathway (Figure 4K).

HPLC fingerprinting was performed to identify the main chemical compounds in the GTS. Fingerprints of the GTS from three batches were identified with satisfactory similarity (Figure 4L), which suggests product stability. Five chemical constituents of the GTS were identified according to the spectrograms and retention time of their standard substances (Figure 4M). The five chemical constituents were measured quantitatively using HPLC: 15.20 ± 0.02 mg/mL chlorogenic acid (from chrysanthemum), 9.16 ± 1.11 mg/mL liquiritin (from liquorice root), 24.89 ± 0.05 mg/mL 5-o-methyIvisammioside (from divaricate saposhniovia root), 65.93 ± 0.26 mg/mL hesperidin (from tangerine peel), and 4.13 ± 0.34 mg/mL 6-gingerol (from ginger).

### 3.4 GTS improves MA-dependent rats by regulating MAPK pathway

**FIGURE 5:**
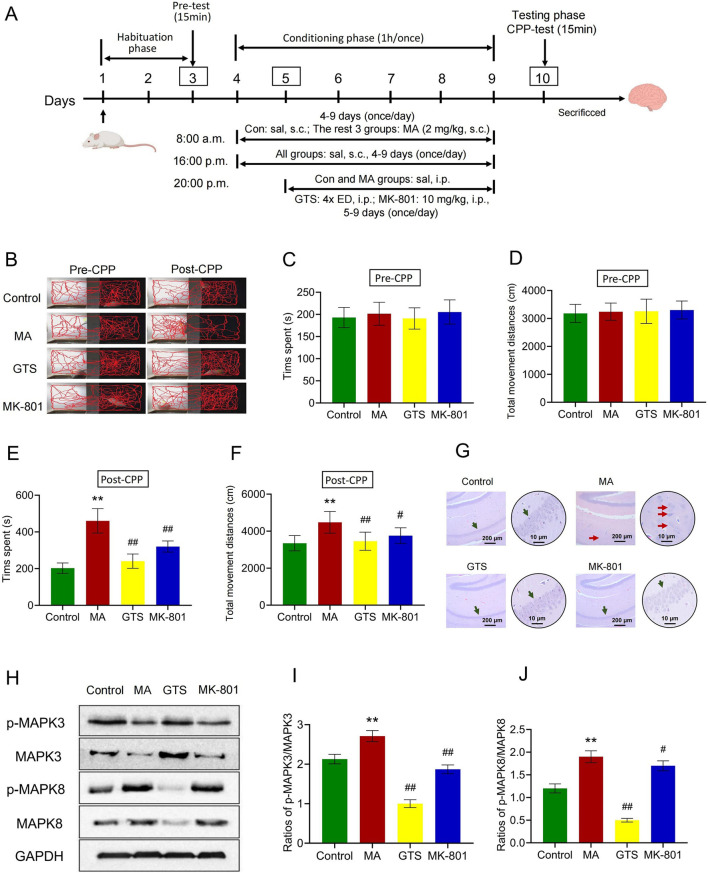
GTS improves the MA-dependent rats by regulating MAPK pathway. **(A)** Schematic protocol of MA-dependent CPP model in rats. **(B)** Activity trajectories of rats in CPP box. Before MA training, the spent time **(C)** and total movement distances **(D)** in the white box of rats, n = 10 per group. After MA training, the spent time **(E)** and total movement distances **(F)** in the white box of rats, n = 10 per group. **(G)** HE staining of pathological changes in rat brain hippocampal tissues. **(H)** The relative expression of p-MAPK3, MAPK3, p-MAPK8 and MAPK8 in rat brain tissues were assessed by Western blot. The ratios of p-MAKP3/MAPK3 **(I)** and p-MAKP8/MAPK8 **(J)** in rat brain tissues, n = 3 per group. Values on the graphs are shown as mean ± SEM, ^**^ *p* < 0.01, ^*^ *p* < 0.05 versus the control group; ^##^ *p* < 0.01, ^#^ *p* < 0.05 versus the MA model group. Pre-CPP, before MA training; Post-CPP, after MA training; Con or Control, control group; MA, MA model group; GTS, GTS treatment group; MK-801, MK-801 positive drug group; sal, saline; ED, equivalent dose.

We further verified the *in vivo* effects of GTS on an MA-dependent rat CPP model (Figure 5A). The movement trajectories of rats were recorded (Figure 5B), and after MA training the movement trajectory of MA model group notably increased compared with the control group. Conversely, the movement trajectories of GTS treatment and MK-801 positive drug groups significantly decreased compared with MA model group. Prior to MA training, there were no significant differences in the time spent (F_3,36_ = 0.83, *p* = 0.48) or total movement distances (F_3,36_ = 0.19, *p* = 0.89) in the white box among all groups (Figures 5C, D). However, following MA training, significant differences were observed in both the time spent (F_3,36_ = 68.94, *p* < 0.01) and total movement distances (F_3,36_ = 11.01, *p* < 0.01) in the white box by rats (Figures 5E, F). The MA model group versus the control group exhibited significant increase in the time spent (*p* < 0.01) and total movement distances (*p* < 0.01) in the white box, indicating the modeling was successful. GTS treatment group demonstrated different reductions in time spent (*p* < 0.01) and total movement distances (*p* < 0.01) in the white box compared with the MA model group. Similarly, MK-801 positive drug group had the same trend as GTS treatment group in time spent (*p* < 0.01) and total movement distances (*p* < 0.05). These findings suggest GTS can mitigate the MA-induced CPP effect.

HE staining showed the pyramidal cells in the hippocampal CA1 region of the control group were tightly arranged, with a clear structure and complete morphology. Compared with the control group, the pyramidal cells in the hippocampal CA1 region of MA model group were arranged loosely, with disordered layers and irregular shapes. Some nuclei were concentrated and deeply stained, cytoplasm was wrinkled, and the damage was significantly aggravated, indicating that MA induction can damage the cerebral hippocampus. The pyramidal cells in the hippocampal CA1 region of GTS treatment and MK-801 positive drug groups versus the MA model group were arranged more tightly and neatly, with a clearer structure and significantly reduced damage, indicating the intervention of GTS has a protective effect on the brain and hippocampus (Figure 5G).

The relative expression of MAPK-related proteins in rat brain tissues were assessed by Western blot (Figure 5H). GTS and MK-801 effectively antagonize the abnormal ratios of p-MAPK3/MAPK3 (F_3,8_ = 114.64, *p* < 0.01) and p-MAPK8/MAPK8 (F_3,8_ = 121.38, *p* < 0.01) induced by MA (Figures 5I, J). The ratios of p-MAPK3/MAPK3 (*p* < 0.01) and p-MAPK8/MAPK8 (*p* < 0.01) were significantly increased in the brain tissues of the MA model group compared to the control group. In contrast, the ratios of p-MAPK3/MAPK3 (*p* < 0.01) and p-MAPK8/MAPK8 (*p* < 0.01) in the GTS treatment group were reduced to varying degrees compared to the MA model group. Similarly, MK-801 positive drug group had the same trend as GTS treatment group in p-MAPK3/MAPK3 (*p* < 0.01) and p-MAPK8/MAPK8 (*p* < 0.05).

### 3.5 GTS improves MA-induced SH-SY5Y cell model by MAPK pathway

**FIGURE 6:**
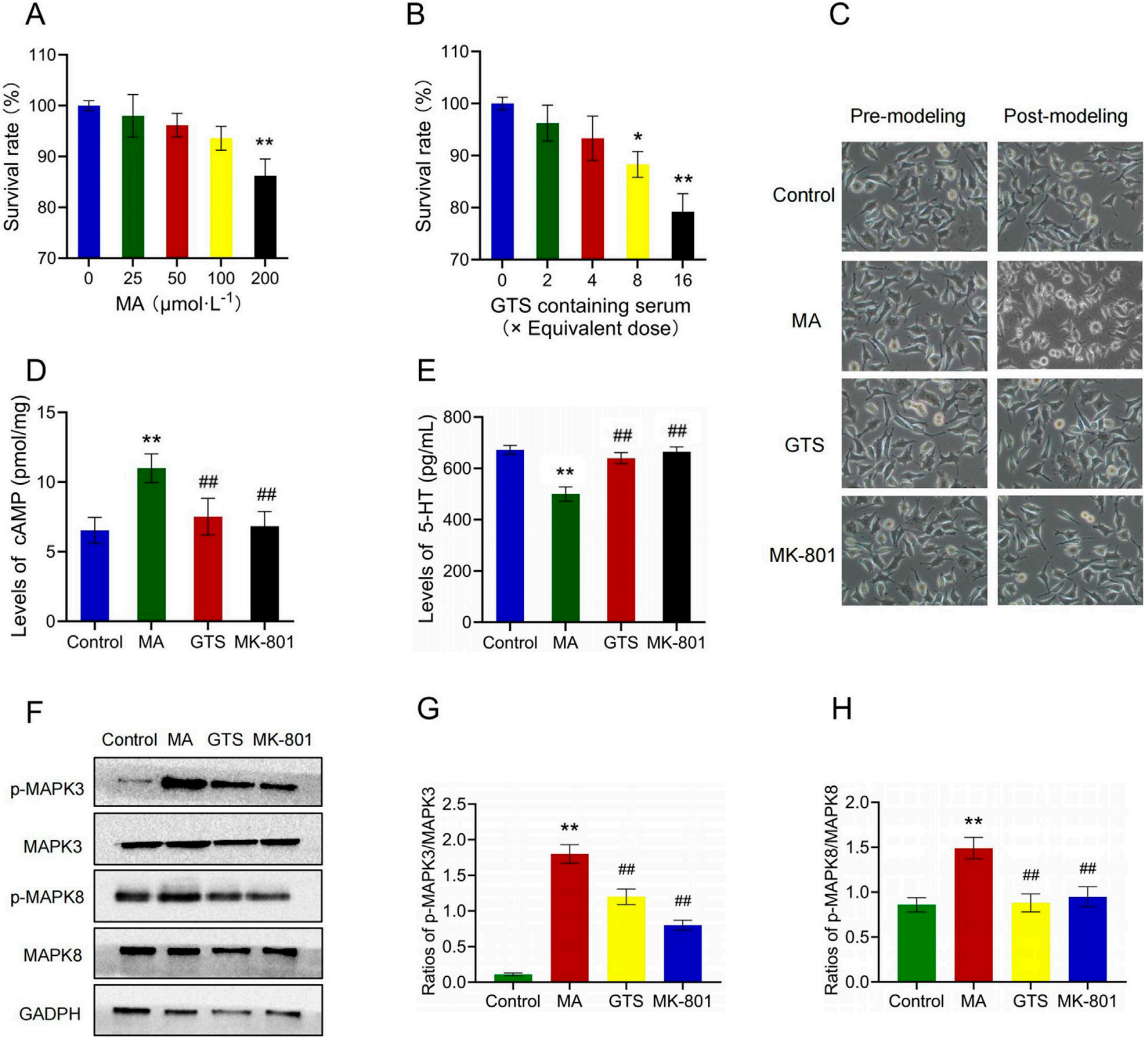
GTS improved the MA-induced SH-SY5Y cell model by MAPK pathway. Effects of MA **(A)** and GTS-containing serum **(B)** on survival rate of SH-SY5Y cells, n = 6 per group **(C)** The morphology of SH-SY5Y cells. The levels of cAMP **(D)** and 5-HT **(E)** in SH-SY5Y cells, n = 6 per group **(F)** The relative expression of p-MAPK3, MAPK3, p-MAPK8 and MAPK8 in SH-SY5Y cells were assessed by Western blot. The ratios of p-MAKP3/MAPK3 **(G)** and p-MAKP8/MAPK8 **(H)** in SH-SY5Y cells, n = 3 per group. Values on the graphs are shown as mean ± SEM, ^**^ *p* < 0.01, ^*^ *p* < 0.05 versus the control group; ^##^ *p* < 0.01, ^#^ *p* < 0.05 versus the MA model group. Control, control group; MA, MA model group; GTS, GTS treatment group; MK-801, MK-801 positive drug group.

The effects of MA (F_4,25_ = 21.43, *p* < 0.01) and GTS-containing serum (F_4,25_ = 35.93, *p* < 0.01) on survival rate of SH-SY5Y cells were assessed by MTT (Figures 6A, B). When the dose of MA reached 200 μmol ·L^−1^, the cell viability significantly decreased (*p* < 0.01) and was below 90%. The maximum safe dose of 100 μmol ·L^−1^ was selected as the modeling dose for MA. When an 8x equivalent dose of the GTS-containing serum was reached, the cell activity decreased significantly (*p* < 0.05). Accordingly, the maximum safe dose (4x equivalent dose) was selected as the therapeutic dose of GTS.

Cell morphology observation showed the cells of each group before modeling were under good growth status with appropriate density, moderate spacing and obvious synapses, and had the classic morphological characteristics of nerve cells. After modeling for 48 h, some cells of the MA model group clustered, with shorter cell protrusions and unclear boundaries. The cell morphology became round, and the supernatant was slightly cloudy, losing the classic morphological features of nerve cells. However, the cell growth status of the rest three groups did not significantly change (Figure 6C).

The levels of cAMP (F_3,20_ = 21.13, *p* < 0.01) and 5-HT (F_3,20_ = 83.87, *p* < 0.01) in SH-SY5Y cells were detected by Elisa (Figures 6D, E). MA model group showed a significant increase in cAMP (*p* < 0.01) and a decrease in 5-HT (*p* < 0.01) compared to the control group. In contrast, the GTS treatment group exhibited a significant decrease in cAMP (*p* < 0.01) and an increase in 5-HT (*p* < 0.01) compared to the MA model group. Similarly, the MK-801 positive drug group had the same trend as GTS treatment group in cAMP (*p* < 0.01) and 5-HT (*p* < 0.01).

The relative expression of MAPK-related proteins in SH-SY5Y cells were assessed by Western blot (Figure 6F). GTS and MK-801 effectively antagonize the abnormal ratios of p-MAPK3/MAPK3 (F_3,8_ = 185.71, *p* < 0.01) and p-MAPK8/MAPK8 (F_3,8_ = 25.78, *p* < 0.01) induced by MA (6G,H). The ratios of p-MAPK3/MAPK3 (*p* < 0.01) and p-MAPK8/MAPK8 (*p* < 0.01) were significantly increased in SH-SY5Y cells of MA model group compared to the control group. In contrast, the ratios of p-MAPK3/MAPK3 (*p* < 0.01) and p-MAPK8/MAPK8 (*p* < 0.01) in the GTS treatment group were reduced to varying degrees compared to the MA model group. Similarly, MK-801 positive drug group had the same trend as GTS treatment group in p-MAPK3/MAPK3 (*p* < 0.01) and p-MAPK8/MAPK8 (*p* < 0.01).

### 3.6 Pharmacokinetics and brain tissue distribution of GTS

**FIGURE 7:**
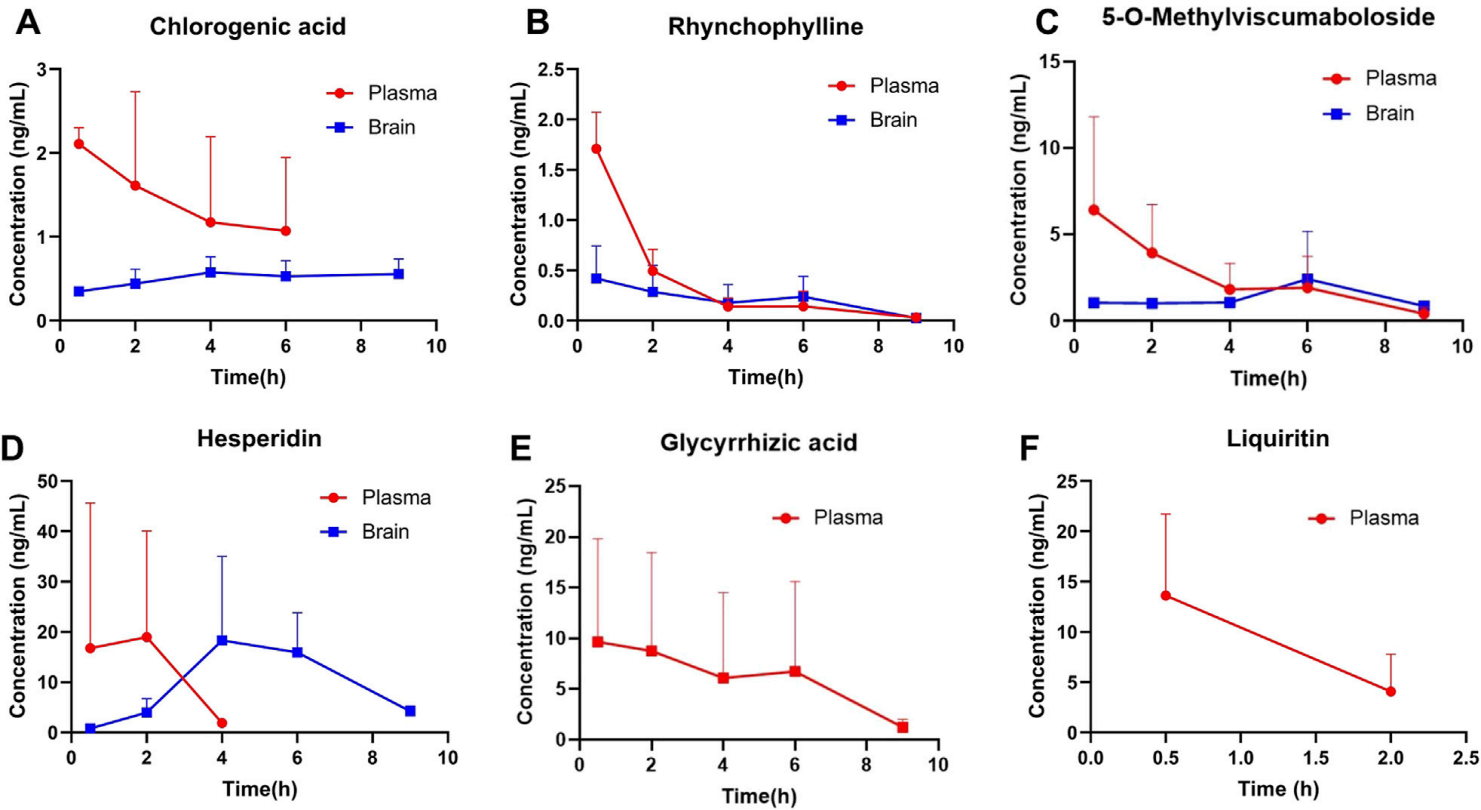
Concentrations of major ingredients in the plasma and brain tissues after oral administration of GTS in rats. **(A)** Chlorogenic acid. **(B)** Rhynchophylline. **(C)** 5-O-Methylviscumaboloside. **(D)** Hesperidin. **(E)** Glycyrrhizic acid. **(F)** Liquiritin.

The six ingredients in mice plasma and brain after validated method for analysis were then subjected to a pharmacokinetic study after an oral administration of GTS. The shapes of the concentration–time curves of chlorogenic acid, rhynchophylline, 5-o-methylviscumaboloside and hesperidin were similar (Figures 7A–D). The concentrations of 4 active ingredients gradually decreased in plasma and slowly increased in brain. The concentrations of chlorogenic acid and hesperidin in brain both peaked after 4 h, and the concentrations of rhynchophylline and 5-o-methylviscumaboloside reached the peak after 6 h. This result may imply that GTS begins to exert therapeutic effects within 4–6 h after gavage of GTS in mice. Glycyrrhizic acid and liquiritin were both detectable in plasma and at high levels, but were undetectable in brain tissues (Figures 7E, F). The above results show some active ingredients of GTS are detectable in both plasma and brain tissues after oral administration of GTS to mice, suggesting the above ingredients play a role in the anti-drug dependence of GTS.

## 4 Discussion

Network pharmacology identified 38 core targets for GTS intervention in DDp, highlighting 14 high-degree targets, including MAPK3 and MAPK8, which are significantly enriched in the MAPK pathway. This finding underscores the critical role of the MAPK pathway in GTS’s therapeutic effects against DDp. PPI analysis constructed a protein network centered on MAPK3, integrating MAPK8 and other MAPK targets. Molecular docking studies revealed stable binding interactions between components of GTS and MAPK3, MAPK8. Furthermore, MA elevated the expression ratios of p-MAPK3/MAPK3 and p-MAPK8/MAPK8 in both rat brain tissues and SH-SY5Y cells. The pivotal roles of MAPK3 (ERK1) and MAPK8 (JNK) in MAPK pathway highlight their involvement in diverse physiological processes, including cell growth, differentiation, and apoptosis (Mordret, 2012). Recent studies increasingly link the MAPK/ERK pathway to long-term potentiation, learning, and memory functions (Feld et al., 2005; Sweatt, 2004). Notably, the ERK pathway is essential for neuronal plasticity associated with memory formation (Adams and Sweatt, 2002; Thomas and Huganir, 2004). Moreover, Wang established the involvement of the JNK pathway in MA-induced apoptosis in SH-SY5Y cells (Wang et al., 2008), prompting extensive research into the phosphorylation status of MAPK3 and MAPK8 to elucidate their roles in the MAPK pathway.

*In vivo* experiments indicated GTS reduced MA-induced CPP effects in rats while offering protection to the hippocampus. GTS alleviated DDp symptoms by regulating MAPK3 and MAPK8. Inhibiting the MAPK pathway has been reported to protect hippocampal neurons (Zhao et al., 2018; Karasewski and Ferreira, 2003; Chen et al., 2016). It is postulated that GTS antagonizes DDp behaviors and exerts neuroprotective effects on hippocampal neurons by restraining MAPK pathway. We established an SH-SY5Y cell model using 100 μmol ·L^−1^ MA for 48 h, demonstrating GTS counteracted aberrant changes in cAMP, 5-HT, and cellular morphology induced by MA, showing therapeutic potential in MA-induced SH-SY5Y models. The doctrine of addictive adaptation provides a biological basis for studying DDp mechanisms via cAMP regulation, where the fundamental molecular basis of DDp lies in cAMP upregulation (Sun et al., 2017). One mechanism of MA involves altering 5-HT release, leading to neuroexcitatory symptoms. Repeated use of addictive substances depletes 5-HT and inhibits its reuptake, causing neurotoxicity (Wang et al., 2019). The MAPK pathway is critical in regulating various cellular activities, including activation, differentiation, and cytokine production. Once inside cells, MA broadly activates the MAPK pathway, contributing to numerous physiological and pathological processes and ultimately causing neuronal damage (Zhu et al., 2018).

Our extraction of GTS ingredients *in vitro* using traditional decoction methods, followed by HPLC analysis of the five major components, then administered the extracted GTS decoction to mice and detected six major components in plasma and brain tissue via HPLC-MS. A validated method for analyzing these ingredients was successfully applied to a pharmacokinetic study post-oral administration. Results showed chlorogenic acid, rhynchophylline, and hesperidin were absorbed into the bloodstream and distributed to brain tissues. Notably, rhynchophylline crossed the blood-brain barrier, exhibiting neuroprotective effects by inhibiting oxidative stress and reducing glutamate-induced excitotoxicity in SH-SY5Y cells (Li et al., 2022). Hesperidin has been reported to mitigate deficiencies in memory, learning, and cognitive abilities inhibited by neuroinflammation-mediated neurodegeneration (Muhammad et al., 2019), with one mechanism being its direct enhancement of synaptic connections between cortical neurons (Matias et al., 2017). Chlorogenic acid was found to prevent neurotoxicity from microglial activation, ultimately improving the survival of dopaminergic neurons (Shen et al., 2012). Our findings suggest that these compounds are crucial ingredients in GTS for treating MA dependence.

During the development of MA dependence, excitatory glutamatergic neurons in the hippocampus directly project to the amygdala, prefrontal cortex, and nucleus accumbens, while also indirectly connecting to dopaminergic neurons in the ventral tegmental area. Research has shown synaptic dysfunction in CA1 region of the hippocampus affects spatial learning abilities in adult rats, leading to memory decline in AD animal models (Sun and Alkon, 2004). Specifically, delayed neuronal death in the CA1 region has been linked to impairments in learning and memory capabilities (Kumaran et al., 2008). Repeated administration of MA damages spatial memory in rats, potentially associated with abnormal ERK1/2 pathway function in the hippocampus (Nagai et al., 2007). Furthermore, clinical studies have reported a significant reduction in gray matter in the amygdala and hippocampus of schizophrenia patients induced by MA (Orikabe et al., 2011). Our findings confirm that MA addiction causes damage to the hippocampus, consistent with previous reports, and that GTS intervention can mitigate this damage.

This study is the first to report that GTS can counteract CPP behavior in MA-dependent rats. Network pharmacology, *in vivo*/*vitro* experiment and pharmacokinetics integrated strategy to reveal pharmacological mechanism of GTS on MA dependence and enabling a comprehensive and precise understanding of the pharmacological mechanisms in other TCM formulations. DDp encompasses both psychological and physical dependence. The CPP model is a classic and reliable method for assessing psychological dependence; however, this study did not include relevant animal experiments related to physical dependence, such as tests for seeking behaviors and withdrawal challenge tests. Additionally, HE staining was only observed CA1 region of hippocampus, without conducting quantitative experiments such as Hoechst staining to count on the slices and did not investigate other brain regions related to DDp, such as the nucleus accumbens, hypothalamus, prefrontal cortex, and amygdala. Furthermore, the focus was consistently on MAPK pathway, neglecting other classical pathways closely associated with DDp, including dopamine neurotransmitter activity and glutamatergic synapses. Addressing these limitations is a priority for future studies involving GTS.

## 5 Conclusion

Network pharmacology revealed MAPK as a critical pathway in GTS’s action against MA dependence, with key GTS compounds binding strongly to MAPK targets, including MAPK3 and MAPK8. HPLC and HPLC-MS detected multiple active GTS compounds *in vitro* and *in vivo*, with four reaching both serum and brain. Experimental validation confirmed the therapeutic effects of GTS and its regulation of MAPK-related proteins in MA-induced *in vitro/vivo* models. This pharmacokinetics-integrated network pharmacology approach offers insights into GTS’s mechanisms and advancing further development of other TCM formulas research.

## Data Availability

The datasets presented in this study can be found in online repositories. The names of the repository/repositories and accession number(s) can be found in the article/supplementary material.

## Glossary

DDp: drug dependence;
CNS: central nervous system
GTS: Gou Teng San
GT: Gou Teng
TCM: traditional Chinese medicine
MA: methamphetamine
AD: Alzheimer’s disease
CPP: conditioned place preference
TCMSP: traditional Chinese medicine system pharmacology
TCMID: traditional Chinese medicine integrated database
PPI: protein-protein interaction
DAVID: database for annotation, visualization and integrated discovery
GO: Gene Ontology
KEGG: Kyoto Encyclopedia of Genes and Genomes
BP: biological process
CC: cellular component
MF: molecular function
OB: oral bioavailability
DL: drug-likeness
BBB: blood–brain barrier
PDB: protein data bank
SD: Sprague-Dawley
NMDA: N-methyl-D-aspartic acid
MK-801: Dizocilpine
PBS: phosphate buffered salt
HE: hematoxylin-eosin
s.c.: subcutaneous injection
i.p.: intraperitoneally injection
Elisa: enzyme-linked immunosorbent assay
ECL: Enhanced chemiluminescence
MAPK: mitogen-activated protein kinase.
